# Blinatumomab for treatment of children with acute lymphoblastic leukaemia in Hong Kong: A cost‐effectiveness analysis

**DOI:** 10.1111/bjh.70582

**Published:** 2026-05-27

**Authors:** Mingjun Rui, Yin Ting Cheung, Hui Zhang, Qiran Wei, Yingcheng Wang, Jiaqi Shi, Chi Kong Li, Joyce H. S. You

**Affiliations:** ^1^ School of Pharmacy, Faculty of Medicine The Chinese University of Hong Kong Hong Kong China; ^2^ Hong Kong Hub of Paediatric Excellence The Chinese University of Hong Kong Hong Kong China; ^3^ Department of Paediatrics, Faculty of Medicine The Chinese University of Hong Kong Hong Kong China; ^4^ Department of Paediatrics and Adolescent Medicine Hong Kong Children's Hospital Hong Kong China

**Keywords:** B‐cell acute lymphoblastic leukaemia, blinatumomab, cost‐effectiveness analysis, Hong Kong

## Abstract

To assess the cost‐effectiveness of blinatumomab plus chemotherapy for paediatric patients from Hong Kong public healthcare provider's perspective, a 10‐year Markov model was designed to simulate outcomes in paediatric patients with newly diagnosed standard‐risk B‐cell ALL at: (1) average risk and (2) high risk of relapse. Three treatment strategies were evaluated: (1) blinatumomab plus chemotherapy as first‐line therapy; (2) blinatumomab plus chemotherapy as second‐line therapy for patients who failed first‐line chemotherapy; (3) chemotherapy alone. Model outcomes included direct medical cost, life‐year gained (LYG), quality‐adjusted life‐year (QALY) gained and incremental cost‐effectiveness ratio (ICER). Comparing to the next less costly strategy, the ICERs of blinatumomab second‐line therapy (10 534 and 12 375 USD/QALY) and blinatumomab first‐line therapy (50 312 and 54 799 USD/QALY) were below the willingness‐to‐pay threshold (162 721 USD/QALY) for high and average risk of relapse groups respectively. One‐way sensitivity analysis found that exponential rates of survival with blinatumomab as first‐line and blinatumomab drug cost were influential factors. In probabilistic sensitivity analysis, blinatumomab first‐line group was the preferred option in 71.4% and 90.3% of simulations in the high and average risk of relapse groups, correspondingly. In conclusion, adding blinatumomab to first‐line therapy appears to be cost‐effective for paediatric patients with newly diagnosed standard‐risk B‐cell ALL.

## BACKGROUND

Acute lymphoblastic leukaemia (ALL) is a haematological malignancy marked by the uncontrolled growth of lymphoblasts.^1^ It is the most common childhood cancer, accounting for 20% of all paediatric malignancies.^2^, ^3^ Advances in chemotherapy‐based regimens over the past decades have markedly improved the survival to about 90% after treatment.^4^ Approximately 10%–15% of patients still experience relapse after chemotherapy.^5^

Blinatumomab is a bispecific T‐cell engager that targets CD19 on B lymphoblasts and CD3 on the normal T cells, inducing T‐cell–mediated cytotoxicity against leukaemic cells. The European Medicines Agency approved blinatumomab for paediatric patients aged 1 year or older with Philadelphia chromosome‐negative CD19‐positive B‐cell precursor acute lymphoblastic leukaemia who have relapsed or refractory disease, as well as for paediatric patients with high‐risk first relapsed Philadelphia chromosome‐negative CD19‐positive B‐cell precursor acute lymphoblastic leukaemia as part of consolidation therapy.^6^ It was also approved by the Food and Drug Administration for paediatric patients aged 1 month and older with CD19‐positive, Philadelphia chromosome–negative B‐cell ALL in the consolidation phase of multiphase chemotherapy.^7^

Recently, the Children's Oncology Group (COG) AALL1731 trial evaluated the addition of blinatumomab to standard chemotherapy in newly diagnosed paediatric patients with standard‐risk B‐cell ALL.^8^ This phase 3 randomized controlled trial enrolled 1440 children aged 1–10 years, demonstrating that the addition of blinatumomab to standard chemotherapy significantly improved disease‐free survival (difference in restricted mean survival time, 72 days [95% CI: 36–108, *p* < 0.001]). The estimated 3‐year cumulative incidence of relapse was lower in both the average‐risk (2.5% vs. 9.8%) and high‐risk (4.3% vs. 14.4%) subgroups treated with blinatumomab and chemotherapy versus chemotherapy alone.^8^ The AALL1731 findings indicate that adding blinatumomab to first‐line chemotherapy improves disease‐free survival in children with standard‐risk B‐cell ALL (at average or high risk of relapse). Previous cost‐effectiveness analyses of blinatumomab focused on relapsed or refractory B‐cell ALL,^9^, ^10^, ^11^ and no study has assessed the cost‐effectiveness of blinatumomab as a component of first‐line therapy in newly diagnosed standard‐risk paediatric patients. The present study aimed to evaluate the potential cost‐effectiveness of blinatumomab plus chemotherapy as first‐line therapy, second‐line therapy and chemotherapy alone for newly diagnosed B‐cell ALL from the perspective of the Hong Kong public healthcare provider.

## METHODS

### Model design

**FIGURE 1:**
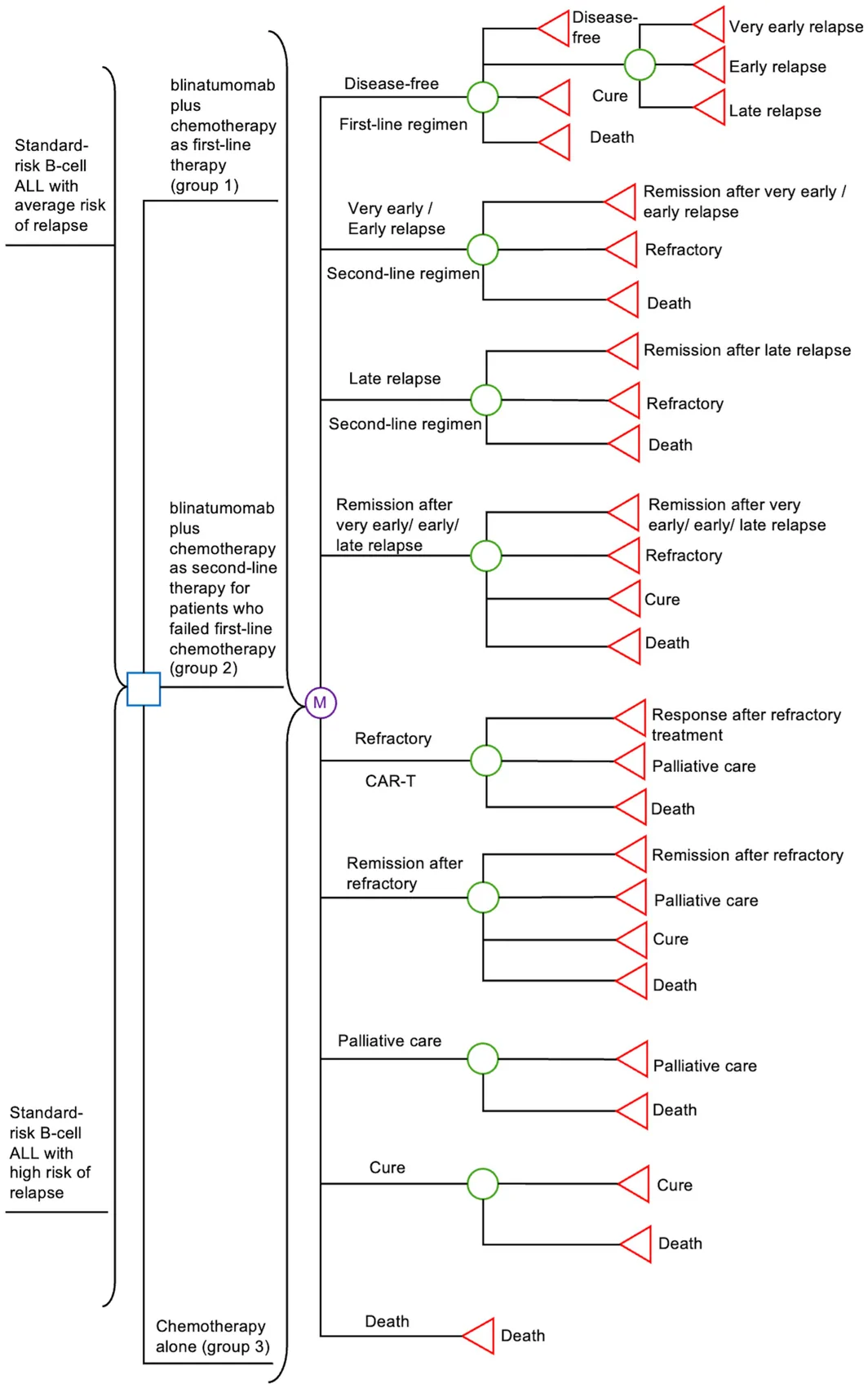
Simplified Markov model structure of acute lymphoblastic leukaemia; the figure illustrates patients' transition between health states, including disease‐free, relapse (very early, early and late), remission after relapse (very early, early and late), refractory, remission after refractory palliative care and death; Group 1: Blinatumomab plus chemotherapy as first‐line therapy, chemotherapy (followed by HSCT) as second‐line therapy; Group 2: Chemotherapy as first‐line therapy, blinatumomab plus chemotherapy (followed by HSCT) as second‐line therapy; Group 3: Chemotherapy as first‐line therapy, chemotherapy with HSCT as second‐line therapy. HSCT, haematopoietic stem cell transplantation.

A Markov model (Figure 1) was constructed to simulate outcomes of two hypothetical cohorts of paediatric patients with newly diagnosed standard‐risk B‐cell ALL at: (1) average risk and (2) high risk of relapse. Three treatment strategies were evaluated in the present model: (1) blinatumomab plus chemotherapy as first‐line therapy (blinatumomab first‐line group); (2) blinatumomab plus chemotherapy as second‐line therapy for patients who failed first‐line chemotherapy (blinatumomab second‐line group); (3) chemotherapy alone (chemotherapy group). The model time horizon was 10 years (with 28‐day cycle) to allow adequate time for estimation of the outcome measures, including direct medical cost, life‐year gained (LYG) and quality‐adjusted life‐year (QALY) gained by each treatment strategy.

All newly diagnosed standard‐risk B‐cell ALL patients entered the model at the health state of disease‐free. Details of regimens for patients with average/high risk of relapse in the blinatumomab first‐line group, blinatumomab second‐line group and chemotherapy group are provided in the Supplementary Materials. Details of the chemotherapy were shown in Tables S1 and S2.

### Clinical inputs

The clinical model inputs are listed in Table 1. The strategy of searching inputs was shown in the Supplementary Materials. The baseline age input for the model was set at 4 years, consistent with the median age of patients in the AALL1731 trial.^8^ The model estimated the transition probabilities of patients who remain in the disease‐free state using the disease‐free survival (DFS) curves from the AALL1731 trial.^8^ The transition probabilities from the disease‐free state to death were estimated from overall survival (OS) curves from the AALL1731 trial.^8^ Separate DFS and OS curves were fitted for patients with an average risk of relapse and for those with a high risk of relapse. Transition probabilities from (very early, early and late) relapse to refractory, and from remission (after very early, early and late relapse) to refractory state were derived from the event‐free survival (EFS) curves reported in the Hong Kong ALL‐Relapse 2000 study, and the probabilities of death from relapse and remission (after relapse) were obtained from the OS curves reported in the same study.^12^ Transition probabilities from refractory (and remission after refractory) to palliative care were derived from the EFS curves reported in the ELIANA study, and the probabilities of refractory (and remission after refractory) to death were obtained from the OS curves reported in the same study.^13^

**TABLE 1:** Model input parameters used in the Markov model.

Parameters	Base‐case value	Range for sensitivity analysis	Distribution	Reference
Clinical inputs				
Age of SR B‐cell ALL patients (years)	4	1–10	Lognormal	8
DFS of blinatumomab plus chemotherapy as first‐line therapy for SR B‐cell ALL				
Exponential rate				
High risk of relapse	0.001725	0.00098–0.003038	Lognormal	8
Average risk of relapse	0.000702	0.000351–0.001403	Lognormal	8
OS of blinatumomab plus chemotherapy as first‐line therapy for SR B‐cell ALL				
Exponential rate				
High risk of relapse	0.000984	0.000492–0.001968	Lognormal	8
Average risk of relapse	0.0000744	0.0000105–0.000528	Lognormal	8
DFS of chemotherapy alone as first‐line therapy for SR B‐cell ALL				
Exponential rate				
High risk of relapse	0.003419	0.002404–0.004861	Lognormal	8
Average risk of relapse	0.00201	0.001369–0.002952	Lognormal	8
OS of chemotherapy alone as first‐line therapy for SR B‐cell ALL				
Exponential rate				
High risk of relapse	0.001033	0.000556–0.00192	Lognormal	8
Average risk of relapse	0.000523	0.00025–0.001098	Lognormal	8
EFS of second‐line treatment for very early relapse				
Gompertz rate	0.14385	0.04883–0.42379	Cholesky decomposition	12
Gompertz shape	−0.16204	−0.3145‐(−0.00958)		12
OS of second‐line treatment for very early relapse				
Gompertz rate	0.0506	0.0171–0.1495	Cholesky decomposition	12
Gompertz shape	−0.0576	−0.1196‐0.0045		12
EFS of second‐line treatment for early relapse				
Gompertz rate	0.01915	0.00817–0.04486	Cholesky decomposition	12
Gompertz shape	−0.03728	−0.06372‐(−0.01083)		12
OS of second‐line treatment for early relapse				
Gompertz rate	0.011188	0.004509–0.027757	Cholesky decomposition	12
Gompertz shape	−0.020459	−0.040589‐(−0.000328)		12
EFS of second‐line treatment for late relapse				
Exponential rate	0.00619	0.002–0.01919	Lognormal	12
OS of second‐line treatment for late relapse				
Exponential rate	0.00344	0.00086–0.01375	Lognormal	12
Hazard ratio for blinatumomab as second‐line therapy				
DFS	0.7	0.46–1.1	Lognormal	14
OS	0.54	0.32–0.92	Lognormal	14
EFS of CAR‐T				
Gompertz rate	0.0597	0.0372–0.0957	Cholesky decomposition	13
Gompertz shape	−0.03728	−0.06372‐(−0.01083)		13
OS of CAR‐T				
Gompertz rate	0.02178	0.01203–0.03943	Cholesky decomposition	13
Gompertz shape	−0.03465	−0.06976‐0.00045		13
Transition probability per 28‐day cycle				
From palliative care to death	0.0807	0.05460–0.1190	Beta	15
Age‐specific natural mortality	0.00009244–0.03423	‐	‐	16
Utility/Disutility inputs				
Utility				
Cure	0.9	0.72–1	Beta	19
Disease‐free	0.87	0.83–0.91	Beta	19
Response	0.87	0.83–0.91	Beta	19
Relapse	0.59	0.49–0.86	Beta	20,21
Refractory	0.59	0.49–0.86	Beta	20,21
Palliative care	0.3	0.24–0.36	Beta	20,21
Disutility				
Seizure	−0.04	−0.032–(−0.048)	Beta	22
Peripheral neuropathy	−0.36	−0.288–(−0.432)	Beta	23
Febrile neutropenia	−0.09	−0.072–(−0.108)	Beta	24
Sepsis or catheter‐related infection	−0.218	−0.174–(−0.262)	Beta	25
Other infection	−0.218	−0.174–(−0.262)	Beta	25
Pancreatitis	−0.17	−0.136–(−0.204)	Beta	26
Allergic reaction	−0.09	−0.072–(−0.108)	Beta	27
Thrombosis	−0.029	−0.023–(−0.035)	Beta	28
Mucositis	−0.084	−0.067–(−0.101)	Beta	30
Cytokine release syndrome	−0.23	−0.184–(−0.276)	Beta	29
Bilirubin increased	−0.03	−0.024–(−0.036)	Beta	31
Tumour lysis syndrome	−0.218	−0.174–(−0.262)	Beta	25
Acute GVHD	−0.467	−0.374–(−0.560)	Beta	23
Incidence of serious adverse event				
Blinatumomab + chemotherapy				
Seizure	1.1%	0.9%–1.3%	Beta	8
Peripheral neuropathy	0.6%	0.5%–0.7%	Beta	8
Febrile neutropenia	47.0%	37.6%–56.4%	Beta	8
Sepsis or catheter‐related infection	14.8%	11.8%–17.8%	Beta	8
Other infection	32.8%	26.2%–39.4%	Beta	8
Pancreatitis	0.9%	0.7%–1.1%	Beta	8
Allergic reaction	2.8%	2.2%–3.4%	Beta	8
Mucositis	13.1%	10.5%–15.7%	Beta	8
Thrombosis	0.3%	0.2%–0.4%	Beta	8
Bilirubin increased	7.7%	6.2%–9.2%	Beta	8
Chemotherapy alone				
Seizure	1.9%	1.5%–2.3%	Beta	8
Peripheral neuropathy	2.4%	1.9%–2.9%	Beta	8
Febrile neutropenia	39.6%	31.7%–47.5%	Beta	8
Sepsis or catheter‐related infection	5.1%	4.1%–6.1%	Beta	8
Other infection	26.3%	21.0%–31.6%	Beta	8
Pancreatitis	1.1%	0.9%–1.3%	Beta	8
Allergic reaction	7.2%	5.8%–8.6%	Beta	8
Mucositis	15.4%	12.3%–18.5%	Beta	8
Thrombosis	0.5%	0.4%–0.6%	Beta	8
Bilirubin increased	7.7%	6.2%–9.2%	Beta	8
Tumour lysis syndrome in CAR‐T	5.0%	4.0%–6.0%	Beta	13
Acute GVHD proportion in HSCT population	62.5%	40%–80%	Beta	17,18
Costs inputs				
Cost per phase (USD, including drug and non‐drug costs)				
Induction (one cycle)	4836	3869‐5803	Gamma	8,34
Consolidation of average risk (one cycle)	1420	1136‐1703	Gamma	8,34
Interim maintenance I (two cycles, average risk of relapse)	2283	1826‐2740	Gamma	8,34
Delayed intensification (two cycles, average risk of relapse)	7226	5781‐8672	Gamma	8,34
Interim maintenance II (two cycles, average risk of relapse)	2305	1844‐2766	Gamma	8,34
Consolidation (two cycles, high risk of relapse)	12 661	10 129‐15 194	Gamma	8,34
Interim maintenance I (two cycles, high risk of relapse)	2553	2042‐3063	Gamma	8,34
Delayed intensification (two cycles, high risk of relapse)	10 608	8487‐12 730	Gamma	8,34
Interim maintenance II (two cycles, high risk of relapse)	5348	4278‐6417	Gamma	8,34
Maintenance (three cycles)	5681	4545‐6817	Gamma	8,34
Very early relapse (per episode)	91 455	73 164–109 746	Gamma	12,34
Early relapse (per episode)	83 822	67 058‐100 587	Gamma	12,34
Late relapse (per episode)	53 931	43 145‐64 717	Gamma	12,34
Car‐T (per episode)	370 575	296 460‐444 690	Gamma	13,34
HSCT (per episode)	60 039	48 031‐72 047	Gamma	12,32–34
Blinatumomab (per cycle)	11 913	9368‐25 910	Gamma	8,14,34
Chemotherapy alone (per cycle)	1409	1127‐1690	Gamma	8,14,34
Palliative care (per cycle)	6426	5141‐7712	Gamma	15,34
Cure (per cycle)	12	9–14	Gamma	34
Serious adverse event cost (USD, including drug and non‐drug costs)				
Seizure	3278	2623‐3934	Gamma	34,35
Peripheral neuropathy	1311	1049‐1573	Gamma	34,36
Febrile neutropenia	2789	2232‐3347	Gamma	34,37
Sepsis or catheter‐related infection	11 531	9225‐13 837	Gamma	34,38
Other infection	8990	7192‐10 789	Gamma	34,38
Pancreatitis	5245	4196‐6294	Gamma	34,39
Thrombosis	1967	1573‐2360	Gamma	34,40
Allergic reaction	656	525–787	Gamma	34,41
Mucositis	2622	2098‐3147	Gamma	34,42
Cytokine release syndrome	4910	3928‐5892	Gamma	30,34
Bilirubin increased	656	525–787	Gamma	34,43
Tumour lysis syndrome	6555	5244‐7866	Gamma	34,44
Acute GVHD	24 264	19 411‐29 117	Gamma	34,45

*Note*: Base‐case value indicates the value of parameter applied in the base‐case analysis. Range for sensitivity analysis indicates the lower and upper limits applied in variation of the parameter value in one‐way sensitivity analysis. Distribution indicates the probability distribution assigned to each parameter in probabilistic sensitivity analysis.

Abbreviations: 1 USD, 7.78 HKD; DFS, disease‐free survival; EFS, event‐free survival; GVHD, Graft‐versus‐host disease; HR, hazard ratio; HSCT, haematopoietic stem cell transplantation; OS, overall survival.

The transition probabilities of blinatumomab plus chemotherapy as second‐line therapy in the model were estimated using parametric survival functions fitted to the EFS and OS curves in the Hong Kong ALL Relapse 2000 study (using a proportional hazards parametric survival model), with the hazard ratios (for DFS and OS of blinatumomab plus chemotherapy versus chemotherapy alone as the second‐line therapy) applied to the chemotherapy survival functions to generate the corresponding transition probabilities. Hazard ratios (HRs) for DFS and OS of blinatumomab plus chemotherapy versus chemotherapy alone as the second‐line therapy were obtained from the findings reported by the AALL1331 trial.^14^

Transition probabilities from palliative care to death were obtained from a retrospective study (*n* = 325) that reported 2‐year survival outcomes in children with multiple relapses of B‐cell ALL.^15^ The transition probability of death for patients who entered the cure state was based on age‐specific mortality rates from the Hong Kong population.^16^

Serious adverse events (SAE) of grade ≥3 were included. The types and rates of SAE were obtained from the AALL1731 and ELIANA study.^8^, ^13^ Acute graft‐versus‐host disease (GVHD) proportion in patients with haematopoietic stem cell transplantation (HSCT) was obtained from the report by an international GVHD research consortium and a retrospective single‐centre outcome study (*n* = 347) on patients undergoing allogeneic transplantation for a median follow‐up of 76 months.^17^, ^18^

### Utility inputs

The utility inputs are shown in Table 1. Utilities of cure, disease‐free and remission were obtained from a prospective cohort study (*n* = 491) that measured health‐related quality of life in children with acute lymphoblastic leukaemia using the Health Utilities Index across different phases of treatment.^19^ The utility values for relapse, refractory and palliative care were obtained from two health economic evaluations of chimeric antigen receptor T‐cell (CAR‐T) therapy in paediatric relapsed or refractory B‐cell ALL.^20^, ^21^ The disutility values of treatment‐related SAE were derived from quality‐of‐life and cost‐effectiveness studies.^22^, ^23^, ^24^, ^25^, ^26^, ^27^, ^28^, ^29^, ^30^, ^31^ The cumulative QALY were discounted annually at a rate of 3% to current year.

### Costs inputs

All cost inputs used in the model are listed in Table 1. The cost analysis was conducted on direct medical costs from the perspective of the Hong Kong public healthcare provider and included all direct medical costs associated with treatment. Treatment costs covered all first‐line therapies during the disease‐free state, including induction, consolidation, interim maintenance, delayed intensification and maintenance for patients with average and high risk of relapse. Treatment costs also included costs during relapse (very early relapse (chemotherapy, HSCT), early relapse (chemotherapy, HSCT), late relapse (chemotherapy)), refractory states (CAR‐T, palliative care) and cure state (specialty outpatient care). Estimates of resource utilization for each health state were based on clinical studies and health economic evaluations.^8^, ^12^, ^14^, ^15^, ^32^, ^33^ Drug costs were local acquisition cost. Unit costs for medical services were obtained from the Hospital Authority fees and charges.^34^ SAE management costs were estimated by combining the duration of hospitalization reported by retrospective cohort studies, real‐world database analyses and observational clinical studies with the corresponding unit costs.^30^, ^35^, ^36^, ^37^, ^38^, ^39^, ^40^, ^41^, ^42^, ^43^, ^44^, ^45^ The cumulative costs were discounted to the current year with an annual rate of 3%.

### Cost‐effectiveness and sensitivity analyses

The analyses were performed using TreeAge Pro 2025 (TreeAge Software Inc., Williamstown, MA) and Microsoft Excel 16.9 (Microsoft Corporation, Redmond, WA, USA). When a treatment option yielded more QALY at higher cost versus an alternative, the incremental cost‐effectiveness ratio (ICER) of the more effective option was calculated by the equation: Δcost/ΔQALY. A treatment option was cost‐effective if the ICER was less than the WTP threshold. Of all strategies with an ICER (vs. next‐less‐costly‐option) below WTP threshold, the strategy with the highest QALY gain was accepted as the preferred cost‐effective strategy. The 3 × gross domestic product (GDP) per capita of Hong Kong (162 721 USD/QALY) was applied as the willingness‐to‐pay (WTP) threshold.^46^

A one‐way sensitivity analysis was conducted to assess the robustness of base‐case results to variation of each model parameter and to identify threshold value of influential parameters (if any). All the parameters were varied over the 95% confidence interval, or upper and lower limits, if available. Otherwise, a range of variation by ±20% of the base‐case value was used. The probabilistic sensitivity analysis was performed using 10 000 times Monte Carlo simulation. Scatterplots were used to present the findings of the probabilistic sensitivity analysis. The probability of each strategy being accepted as cost‐effective was determined over a range of WTP values from 0 to 162 721 USD/QALY per QALY (3× GDP per capita) in the acceptability curves.

## RESULTS

### Base‐case analysis

Expected direct medical costs, QALY, LYG, incremental costs and incremental QALY in patients with high risk of relapse and those with average risk of relapse are shown in Table 2 respectively.

**TABLE 2:** Base‐case analysis of direct medical cost, QALY and LYG, increment cost, incremental QALY and ICER.

Strategy	Total direct medical cost (USD)	Total QALY	Total LYG	Versus chemotherapy group			Versus the next‐less‐costly strategy		
				Incremental cost (USD)	Incremental QALY	ICER (USD/QALY)	Incremental cost (USD)	Incremental QALY	ICER (USD/QALY)
In standard‐risk B‐cell ALL patients with high risk of relapse									
Chemotherapy group	108 809	6.51	8.98	—	—	—	—	—	—
Blinatumomab second‐line group	109 772	6.60	9.12	963	0.09	10 534	963	0.09	10 534
Blinatumomab first‐line group	118 027	6.77	9.26	9217	0.26	36 080	8255	0.16	50 312
In standard‐risk B‐cell ALL patients with average risk of relapse									
Chemotherapy group	77 820	6.98	9.41	—	—	—	—	—	—
Blinatumomab second‐line group	78 480	7.03	9.50	660	0.05	12 375	660	0.05	12 375
Blinatumomab first‐line group	91 880	7.28	9.83	14 060	0.30	47 203	13 400	0.24	54 799

*Note*: Total direct medical cost, total quality‐adjusted life‐years (QALYs) and total life‐years gained (LYG) are reported for each strategy. Incremental costs, incremental QALYs and incremental cost‐effectiveness ratio (ICER) of each strategy versus chemotherapy alone and versus the next‐less‐costly strategy are reported; 1 USD = 7.78 HKD.

For patients with high risk of relapse (Table 2), the blinatumomab first‐line group gained the highest QALY and LYG at the highest total cost, followed by blinatumomab second‐line and the chemotherapy group. When compared with the chemotherapy group, the ICERs of the blinatumomab second‐line group (10 534 USD/QALY) and the blinatumomab first‐line group (36 080 USD/QALY) were both below the WTP threshold (162 721 USD/QALY per QALY) and accepted as cost‐effective. When compared with the next less costly strategy, the ICERs of the blinatumomab second‐line group (10 534 USD/QALY) and the blinatumomab first‐line group (50 312 USD/QALY) were below the WTP threshold. The blinatumomab first‐line group gained the highest QALY at an ICER < WTP and therefore was the preferred cost‐effective option in patients with high risk of relapse.

For patients with average risk of relapse (Table 2), the blinatumomab first‐line group gained the highest QALY and LYG at the highest total cost, followed by the blinatumomab second‐line group and the chemotherapy group. When compared with the chemotherapy group, the ICERs of the blinatumomab second‐line group (12 375 USD/QALY) and the blinatumomab first‐line group (47 203 USD/QALY) were both below the WTP threshold (162 721 USD/QALY per QALY) and accepted as cost‐effective. When compared with the next less costly strategy, the ICERs of the blinatumomab second‐line group (12 375 USD/QALY) and the blinatumomab first‐line group (54 799 USD/QALY) were below the WTP threshold. The blinatumomab first‐line group gained the highest QALY at an ICER < WTP and, therefore, was also the preferred cost‐effective option in patients with average risk of relapse.

### Sensitivity analysis

The influential factors with threshold values identified in one‐way sensitivity analysis to change the preferred cost‐effective option from blinatumomab first‐line group to blinatumomab second‐line group are shown in Table 3. For patients with high risk of relapse, five influential factors were found: exponential rates of OS with first‐line blinatumomab, OS with first‐line chemotherapy, DFS with first‐line blinatumomab and DFS with first‐line chemotherapy and cost of blinatumomab. For patients with average risk of relapse, two influential factors were identified: exponential rate of DFS with first‐line blinatumomab and cost of blinatumomab.

**TABLE 3:** Threshold values of influential parameters in one‐way sensitivity analysis.

Influential parameters	Base‐case value	Threshold value^a^	Change of preferred cost‐effective strategy when the influential parameters crossed the threshold value^b^
SR B‐cell ALL patients with high risk of relapse			
Exponential rate of OS of blinatumomab plus chemotherapy as first‐line therapy	0.000984	>0.001374^c^	Blinatumomab second‐line group
Exponential rate of DFS of blinatumomab plus chemotherapy as first‐line therapy	0.001725	>0.002253^d^	Blinatumomab second‐line group
Exponential rate of OS of chemotherapy alone as first‐line therapy	0.001033	<0.000626^c^	Blinatumomab second‐line group
Exponential rate of DFS of chemotherapy alone as first‐line therapy	0.003419	<0.002794^d^	Blinatumomab second‐line group
Cost of blinatumomab per cycle	11 913	>21 984	Blinatumomab second‐line group
SR B‐cell ALL patients with average risk of relapse			
Exponential rate of DFS of blinatumomab plus chemotherapy as first‐line therapy	0.000702	>0.001380^d^	Blinatumomab second‐line group
Cost of blinatumomab per cycle	11 913	>25 831	Blinatumomab second‐line group

^a^ The preferred cost‐effective strategy changed when the value of influential parameters crossed the threshold value.

^b^ Base‐case preferred cost‐effective strategies: Blinatumomab first‐line group in SR B‐cell ALL patients with high risk of relapse, blinatumomab first‐line group in SR B‐cell ALL patients with average risk of relapse.

^c^ Higher OS exponential rate indicates faster occurrence of death.

^d^ Higher DFS exponential rate indicates faster relapse.

The scatter plots of the increment costs and QALY gained by the blinatumomab first‐line group and second‐line group versus chemotherapy group for patients with high risk and average risk of relapse are shown in Figure S1A–D.

**FIGURE 2:**
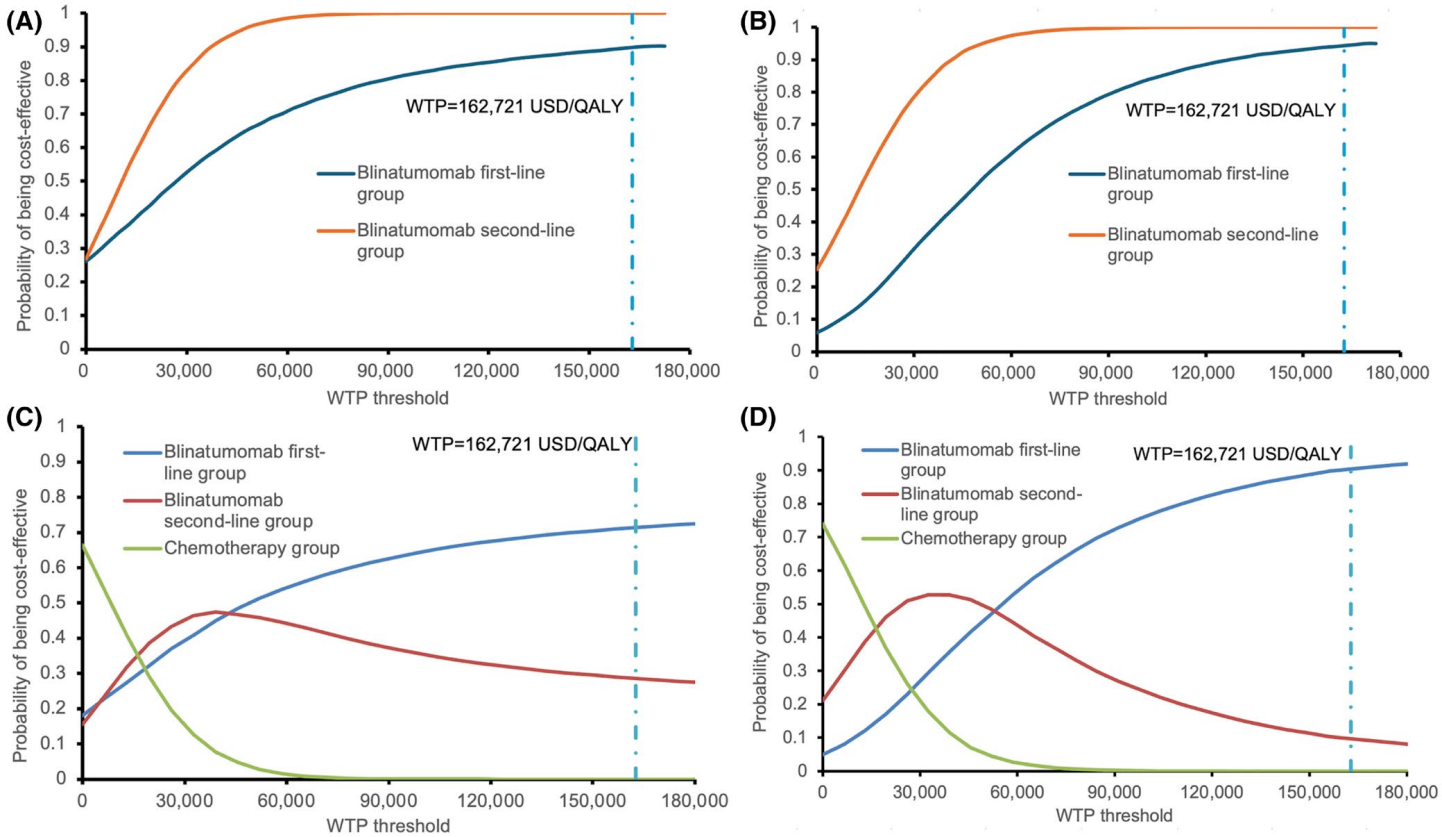
Cost‐effectiveness acceptability curves of blinatumomab first‐line group and blinatumomab second‐line group (each vs. chemotherapy alone) in the ALL patients with high risk of relapse (A); blinatumomab first‐line group and blinatumomab second‐line group (each vs. chemotherapy alone) in the ALL patients with average risk of relapse (B); cost‐effectiveness acceptability curves of all strategies (each vs. the next less costly option) in the ALL patients with high risk of relapse (C); cost‐effectiveness acceptability curves of all strategies (each vs. the next less costly option) in the ALL patients with average risk of relapse (D). ALL, acute lymphoblastic leukaemia; QALY, quality‐adjusted life‐year; WTP, willingness‐to‐pay.

The acceptability curves of the blinatumomab groups, each comparing with the chemotherapy group, are shown in Figure 2A,B. For patients with high risk of relapse (Figure 2A), the blinatumomab first‐line group and the blinatumomab second‐line group were cost‐effective in 89.8% and 100% of iterations, respectively, at WTP = 162 721 USD/QALY. For patients with average risk of relapse (Figure 2B), the blinatumomab first‐line group and the blinatumomab second‐line group was cost‐effective in 94.4% and 100% of simulations, correspondingly, at WTP = 162 721 USD/QALY. The probabilities of each strategy to be the preferred cost‐effective option in patients are shown in Figure 2C,D. For patients with high risk of relapse (Figure 2C), the probabilities to be the preferred cost‐effective option at WTP = 162 721 USD/QALY were 71.4% for the blinatumomab first‐line group, 28.6% for the blinatumomab second‐line group and 0% for the chemotherapy group. For patients with average risk of relapse (Figure 2D), the probabilities to be the preferred cost‐effective option at WTP = 162 721 USD/QALY were 90.3% for the blinatumomab first‐line group, 9.7% for the blinatumomab second‐line group and 0% for the chemotherapy group.

## DISCUSSION

This is the first cost‐effectiveness analysis of blinatumomab plus chemotherapy as first‐line therapy for children newly diagnosed with standard‐risk B‐cell ALL. Base‐case findings showed that blinatumomab, added to both first‐line and second‐line therapy, gained higher QALY and was cost‐effective when compared to chemotherapy alone for newly diagnosed ALL patients (with high and average risk of relapse). The blinatumomab first‐line group was the preferred cost‐effective option for patients with high and average risk of relapse. One‐way sensitivity analysis results showed that higher exponential rates of DFS and OS of blinatumomab plus chemotherapy as first‐line therapy and higher blinatumomab cost (per cycle) were influential parameters of base‐case results. Higher exponential rate of OS represented faster occurrence of mortality, and higher exponential rate of DFS represented faster relapse, and both were the main driving factors of QALY gained. When the exponential rates of OS and DFS of blinatumomab first‐line group varied across the threshold values to faster occurrence of death and relapse, the incremental QALY gained by blinatumomab first‐line group decreased, thus increased the ICER beyond the WTP threshold (and became not accepted as cost‐effective). In addition to lowering QALY gained, the higher exponential rate of DFS of blinatumomab first‐line group also increased total costs when relapse occurred faster, thus increased the ICER beyond the WTP threshold. Increase in the per‐cycle cost of blinatumomab beyond the threshold values led to higher incremental cost for the blinatumomab first‐line group, resulting in ICERs exceeding the WTP threshold and a change of the preferred cost‐effective strategy to blinatumomab second‐line group. The probabilistic sensitivity analysis found that the base‐case results were more robust in the patients with average risk of relapse (90.3% of iterations) than for patients with high risk of relapse (71.4% of iterations).

Previous economic evaluations have compared blinatumomab versus chemotherapy in paediatric patients with relapsed or refractory B‐cell ALL, and blinatumomab was found to be cost‐effective at the WTP thresholds used in different countries.^9^, ^10^, ^11^ A cost‐effectiveness analysis in Mexico evaluated blinatumomab as consolidation therapy for high‐risk first relapse. Blinatumomab gained more life‐years at higher total costs with an ICER of 121 526 Mexican pesos per life‐year gained, lower than Mexico national WTP of 200 000 to 600 000 Mexican pesos (USD 1 = 17.92 Mexican pesos). The results were mainly influenced by the proportion of patients receiving allogeneic haemopoietic stem cell transplantation, dosing of blinatumomab and vial sharing assumption.^9^ A cost‐utility analysis in Iran assessed blinatumomab as second‐line therapy compared with consolidation chemotherapy and found that blinatumomab gained more QALY at higher costs with an ICER of 4160 USD per QALY gained, below the Iran WTP threshold of 4210 USD per QALY. The cost of blinatumomab, the utility values for pre‐progression and post‐progression states and the probability of allogeneic haemopoietic stem cell transplantation were the main influential factors.^10^ A health evaluation in France also compared blinatumomab with consolidation chemotherapy for high‐risk first relapse and found that blinatumomab gained more life‐years at higher cost, with an ICER of 52 298 euros per QALY remaining within the WTP threshold of 150 000 euros per QALY (USD 1 = 0.85 euros). The influential factors were the proportion of allogeneic haemopoietic stem cell transplantation and dosing of blinatumomab.^11^ The present study assessed cost‐effectiveness of blinatumomab as first‐line therapy, and our findings were consistent with prior health economic evaluations that clinical effectiveness of blinatumomab plus chemotherapy as first‐line therapy (as represented by exponential rates on DFS and OS) and blinatumomab drug cost were the key influential factors on the cost‐effectiveness of adding blinatumomab to standard treatment.

The present findings have several clinical implications. The base‐case results showed that adding blinatumomab to first‐line therapy in the treatment pathway gained higher LYG and QALY at an additional cost (acceptable by WTP threshold) for newly diagnosed standard‐risk B‐cell ALL patients (with average risk and high risk of relapse). This suggested that the initiation timing of blinatumomab therapy is an important determinant of long‐term cost‐effectiveness outcomes. Our results also highlighted the importance of treatment strategies that reduced relapse in newly diagnosed patients, thus reduced the utilization of second‐line therapy (chemotherapy and HSCT) and downstream refractory disease treatment (CAR‐T). The cost of blinatumomab (per cycle) was a key cost driver to the cost‐effectiveness use of blinatumomab as a first‐line agent. Region‐wide and institutional‐based drug price negotiation is essential to further enhance the cost‐effectiveness of blinatumomab for first‐line treatment of newly diagnosed standard‐risk B‐cell ALL patients.

This study has some limitations. The model was simplified and may not capture all treatment variations in clinical practice. The model included SAEs with severe outcomes ≥grade 3 requiring medical therapy. The costs and QALY loss by less serious adverse events were not included, and the impact of adverse drug events was, therefore, not fully represented. The survival in the present model was extrapolated by parametric survival distribution methods due to the lack of long‐term efficacy evidence. The sensitivity analyses addressed some of these limitations by varying key clinical parameters over wide ranges to allow assessing the impact of model parameter uncertainties on the cost‐effectiveness results. The analysis adopted the healthcare provider's perspective to include direct medical costs, and indirect costs were, therefore, not considered. Indirect costs refer to productivity loss for caregivers due to time spent providing care and productivity loss among patients resulting from premature mortality. The present analysis might have underestimated the cost‐effectiveness of adding blinatumomab to first‐line therapy.

## CONCLUSION

Adding blinatumomab to chemotherapy as first‐line therapy appears to be the preferred cost‐effective strategy of all examined strategies for newly diagnosed standard‐risk B‐cell ALL patients aged 1–10 years with average or high risk of relapse, from the perspective of healthcare providers in Hong Kong. The cost‐effectiveness acceptance of blinatumomab plus chemotherapy as first‐line therapy was subject to first‐line treatment that determined the patient time in disease‐free state and survival and the drug cost of blinatumomab.

## AUTHOR CONTRIBUTIONS

**Mingjun Rui:** Conceptualization; data curation; formal analysis; investigation; methodology; software; validation; writing – original draft. **Qiran Wei:** Data curation; formal analysis; methodology; software; validation; writing – review and editing. **Yin Ting Cheung:** Conceptualization; formal analysis; methodology; writing – review and editing. **Jiaqi Shi:** Data curation; formal analysis; methodology; software; validation; writing – review and editing. **Joyce H. S. You:** Conceptualization; formal analysis; investigation; methodology; resources; software; supervision; validation; writing – review and editing. **Yingcheng Wang:** Data curation; formal analysis; methodology; software; validation; writing – review and editing. **Hui Zhang:** Conceptualization; methodology; formal analysis; writing – review and editing. **Chi Kong Li:** Conceptualization; formal analysis; investigation; methodology; writing – review and editing.

## FUNDING INFORMATION

This work received no funding.

## CONFLICT OF INTEREST STATEMENT

The authors declare no conflict of interests.

## Supporting information


**Table S1.** Details of the chemotherapy regimens in standard‐risk B‐cell ALL with average risk of relapse and high‐risk B‐cell ALL with average risk of relapse.^1^
**Table S2.** Details of the chemotherapy regimens in patients with very early, early and late relapse.^2^
**Figure S1.** Scatter plots of incremental cost and QALY gained in 10 000 Monte Carlo simulations: (A) Blinatumomab first‐line group versus chemotherapy group for patients with high risk of relapse; (B) blinatumomab second‐line group versus chemotherapy group for patients with high risk of relapse; (C) blinatumomab first‐line group versus chemotherapy group for patients with average risk of relapse; (D) blinatumomab second‐line group versus chemotherapy group for patients with average risk of relapse. QALY, quality‐adjusted life‐year; WTP, willingness‐to‐pay.

## Data Availability

Data are provided within the manuscript and supplementary files.
