# Supplementary material for: Blinatumomab for treatment of children with acute lymphoblastic leukaemia in Hong Kong: A cost‐effectiveness analysis
Source: Br J Haematol. 2026 May 27;209(3):1146–57. doi: 10.1111/bjh.70582 (PMC13570225; doi:10.1111/bjh.70582)
Supplement: Supplementary file 1 [file BJH-209-1146-s001.docx]

**Supplementary Materials**

**Methods**

**Regimens for patients with average/high risk of relapse:**

In the blinatumomab first-line group, patients received first-line chemotherapy plus blinatumomab.^1^ Details of the chemotherapy regimens for patients with average/high risk of relapse are provided in **Supplementary Materials Tables S1**. The first-line chemotherapy for patients with average risk of relapse consisted of sequential phases, including induction (28 days), consolidation (28 days), interim maintenance I (56 days), delayed intensification (56 days), interim maintenance II (56 days), and maintenance (84 days). Maintenance therapy continued until two years after the start of post-consolidation therapy.^1^ Patients with high risk of relapse received first-line chemotherapy consisting of sequential phases, including induction (28 days), consolidation (56 days), interim maintenance I (56 days), delayed intensification (63 days), interim maintenance II (56 days), and maintenance (84 days). Maintenance therapy also continued until two years after the start of post-consolidation therapy.^1^ Blinatumomab was administered for two 28-day cycles at a dose of 15 μg/m^2^ per day (maximum 28 μg per day), before and after the first interim maintenance phase of therapy.^1^ Patients who experienced relapse after first-line therapy were categorized according to the timing of relapse as very early relapse, early relapse, and late relapse. Very early relapse was defined as relapse occurring within 18 months after initial diagnosis. Early relapse was defined as relapse occurring between 18 and 30 months. Late relapse was defined as relapse occurring after more than 30 months. ^2^ Chemotherapy was administered to all relapsed patients according to the HK ALL-Relapse 2000 protocol^2^, which was modified from the BFM relapsed protocol,^3^ followed by hematopoietic stem cell transplantation (HSCT) to those with very early or early relapse. The details of the HK ALL-Relapse 2000 chemotherapy regimens are provided in **Supplementary Materials Tables S2.** ^2^

In the blinatumomab second-line group, patients only received first-line chemotherapy. Those patients who relapsed after first-line therapy were categorized according to the timing of relapse as very early, early, or late relapse. Blinatumomab plus chemotherapy was administered to all relapsed patients, followed by HSCT to those with very early or early relapse. Blinatumomab was initiated after completion of the first cycle of HK ALL-Relapse 2000 chemotherapy protocol, administered as two continuous 28-day cycles (at a dose of 15 μg/m² per day; separated by a 7-day treatment-free interval).^2,4^ In the chemotherapy group, patients only received first-line chemotherapy. For patients who experienced relapse, same second-line therapy (including chemotherapy and HSCT) for the blinatumomab first-line group described above was applied.

In all three treatment arms, patients who received second-line treatment for relapse might achieve remission or remain refractory. Patients who relapsed again after remission were also considered as refractory ALL. Chimeric antigen receptor T-cell (CAR-T) therapy was the subsequent treatment for patients who advanced to refractory ALL. Palliative care was provided to patients who did not achieve remission after CAR-T or relapsed after response to CAR-T. Patients who were disease-free, or achieved remission with treatment for relapse/refractory for 5 years were considered cure.

**Table S1 Details of the chemotherapy regimens in standard-risk B-cell ALL with average risk of relapse and high-risk B-cell ALL with average risk of relapse ^1^**

| **In standard-risk B-cell ALL with average risk of relapse** | | | | | |
| --- | --- | --- | --- | --- | --- |
| **Drug** | **Route** | | **Dose** | **Schedule** | |
| **Induction (28 days)** | | | | | |
| Cytarabine | IT | | Age-based dosing | Day 1 | |
| Dexamethasone | PO | | 3 mg/m², twice daily | Days 1–28 | |
| Vincristine | IV | | 1.5 mg/m² (max 2 mg) | Days 8, 15, 22 | |
| PEG-Asparaginase | IV | | 2,500 IU/m² | Day 4 | |
| Methotrexate | IT | | Age-based dosing | Days 8 and 29 | |
| **Consolidation (28 days)** | | | | | |
| Vincristine | IV | | 1.5 mg/m² (max 2 mg) | Day 1 | |
| Mercaptopurine | PO | | 75 mg/m² daily | Days 1–28 | |
| Methotrexate | IT | | Age-based dosing | Days 1, 8, and 15 | |
| **Interim Maintenance I (56 days)** | | | | | |
| Vincristine | IV | | 1.5 mg/m² (max 2 mg) | Days 1, 11, 21, 31, 41 | |
| Methotrexate | IV | | 100 mg/m², escalate to 500 mg/m² | Days 1, 11, 21, 31, 41 | |
| Methotrexate | IT | | Age-based dosing | Day 31 | |
| **Delayed Intensification (56 days)** | | | | | |
| Dexamethasone | PO | | 5 mg/m², twice daily | Days 1–7 and 15–21 | |
| Vincristine | IV | | 1.5 mg/m² (max 2 mg) | Days 1, 8, 15 | |
| Doxorubicin | IV | | 25 mg/m² | Days 1, 8, 15 | |
| PEG-Asparaginase | IV | | 2,500 IU/m² | Day 4 | |
| Methotrexate | IT | | Age-based dosing | Day 1 | |
| Cyclophosphamide | IV | | 1,000 mg/m² | Day 29 | |
| Thioguanine | PO | | 60 mg/m² daily | Days 29–42 | |
| Cytarabine | IV/SQ | | 75 mg/m² | Days 29–32 and 36–39 | |
| Methotrexate | IT | | Age-based dosing | Day 29 | |
| **Interim Maintenance II (56 days)** | | | | | |
| Vincristine | IV | | 1.5 mg/m² (max 2 mg) | Days 1, 11, 21, 31, 41 | |
| Methotrexate | IV | | Starting dose two-thirds of maximum tolerated dose from Interim Maintenance I, escalate to 50 mg/m² | Days 1, 11, 21, 31, 41 | |
| Methotrexate | IT | | Age-based dosing | Days 1 and 31 | |
| **Maintenance (Cycles of 84 days)** | | | | | |
| Vincristine | IV | | 1.5 mg/m² (max 2 mg) | Day 1 of each cycle | |
| Dexamethasone | PO | | 3 mg/m², twice daily | Days 1–5 of each cycle | |
| Mercaptopurine | PO | | 75 mg/m² daily | Days 1–85 of each cycle | |
| Methotrexate | PO | | 20 mg/m² weekly | Days 8, 15, 22, 29, 36, 43, 50, 57, 64, 72, 78 | |
| Methotrexate | IT | | Age-based dosing | Day 1 of each cycle | |
| **In standard-risk B-cell ALL with high risk of relapse** | | | | | |
| **Drug** | | **Route** | **Dose** | **Schedule** |  |
| **Induction (28 days)** | | | | |  |
| Cytarabine | | IT | Age-based dosing | Day 1 |  |
| Dexamethasone | | PO | 3 mg/m², twice daily | Days 1–28 |  |
| Vincristine | | IV | 1.5 mg/m² (max 2 mg) | Days 8, 15, 22 |  |
| PEG-Asparaginase | | IV | 2,500 IU/m² | Day 4 |  |
| Methotrexate | | IT | Age-based dosing | Days 8 and 29 |  |
| **Consolidation (56 days)** | | | | |  |
| Cyclophosphamide | | IV | 1,000 mg/m² | Days 1 and 29 |  |
| Cytarabine | | IV/SQ | 75 mg/m² | Days 1–4, 8–11, 29–32, 36–39 |  |
| Mercaptopurine | | PO | 60 mg/m² daily | Days 1–14 and 42–56 |  |
| Vincristine | | IV | 1.5 mg/m² (max 2 mg) | Days 15, 43, 50 |  |
| PEG-Asparaginase | | IV | 2,500 IU/m² | Days 15 and 43 |  |
| Methotrexate | | IT | Age-based dosing | Days 1, 8, 15, and 22 |  |
| **Interim Maintenance I (56 days)** | | | | |  |
| Vincristine | | IV | 1.5 mg/m² (max 2 mg) | Days 1, 15, 29, and 43 |  |
| Methotrexate | | IV | 5,000 mg/m² | Days 1, 15, 29, and 43 |  |
| Mercaptopurine | | PO | 25 mg/m² daily | Days 1–14, 15–28, 29–42, 43–56 |  |
| Leucovorin | | PO/IV | 15 mg/m² × 3 | following each high-dose Methotrexate |  |
| Methotrexate | | IT | Age-based dosing | Days 1 and 29 |  |
| **Delayed Intensification (63 days)** | | | | |  |
| Dexamethasone | | PO | 5 mg/m², twice daily | Days 1–7 and 15–21 |  |
| Vincristine | | IV | 1.5 mg/m² (max 2 mg) | Days 1, 8, and 15 |  |
| Doxorubicin | | IV | 25 mg/m² | Days 1, 8, and 15 |  |
| PEG-Asparaginase | | IV | 2,500 IU/m² | Day 4 |  |
| Methotrexate | | IT | Age-based dosing | Day 1 |  |
| Cyclophosphamide | | IV | 1,000 mg/m² | Day 29 |  |
| Thioguanine | | PO | 60 mg/m² daily | Days 29–42 |  |
| Cytarabine | | IV/SQ | 75 mg/m² | Days 29–32 and 36–39 |  |
| Vincristine | | IV | 1.5 mg/m² (max 2 mg) | Days 43 and 50 |  |
| Methotrexate | | IT | Age-based dosing | Days 29 and 36 |  |
| PEG-Asparaginase | | IV | 2,500 IU/m² | Day 43 |  |
| **Interim Maintenance II (56 days)** | | | | |  |
| Vincristine | | IV | 1.5 mg/m² (max 2 mg) | Days 1, 11, 21, 31, and 41 |  |
| Methotrexate | | IV | 100 mg/m², escalate to 500 mg/m² | Days 1, 11, 21, 31, and 41 |  |
| PEG-Asparaginase | | IV | 2,500 IU/m² | Days 2 and 22 |  |
| Methotrexate | | IT | Age-based dosing | Days 1 and 31 |  |
| **Maintenance (Cycles of 84 days)** | | | | |  |
| Vincristine | | IV | 1.5 mg/m² (max 2 mg) | Day 1 of each cycle |  |
| Dexamethasone | | PO | 3 mg/m², twice daily | Days 1–5 of each cycle |  |
| Mercaptopurine | | PO | 75 mg/m² daily | Days 1–85 of each cycle |  |
| Methotrexate | | PO | 20 mg/m² weekly | Days 8, 15, 22, 29, 36, 43, 50, 57, 64, 72, and 78 |  |
| Methotrexate | | IT | Age-based dosing | Day 1 of each cycle and Day 29 of cycles 1 and 2 |  |
| **Blinatumomab plus chemotherapy (Cycles of 28 days)** | | | | |  |
| Dexamethasone | | PO | 5 mg/m² (max 20 mg) | Prior to start of blinatumomab |  |
| Blinatumomab | | IV | Continuous infusion, 15 μg/m²/day | Days 1–28 |  |
| Methotrexate | | IT | Age-based dosing | Day 1 |  |

IT – Intrathecal; IV – Intravenous; PO – Oral; SQ – subcutaneous; Cytarabine IT – age 1-1.99 years: 30 mg; 2-2.99 years: 50 mg; >=3 years: 70 mg; Methotrexate IT – age 1-1.99 years: 8 mg; 2-2.99 years: 10 mg; 3-8.99 years: 12 mg; >= 9 years: 15 mg; Maintenance cycles continued until two years after the start of post-Consolidation therapy

**Table S2 Details of the chemotherapy regimens in patients with very early, early and late relapse^2^**

| **In patients with very early relapse** | | | |
| --- | --- | --- | --- |
| **Drug** | **Route** | **Dose** | **Schedule** |
| **Cycle 1** | | | |
| Dexamethasone | PO | 6 mg/m²/day | Days 1–5, 8–13, 15–20 |
| Triple intrathecal chemotherapy | IT | — | Day 1,12 |
| Vincristine | IV | 1.5 mg/m² (max 2 mg) | Day 1, 8 and 13 |
| Cytarabine | IV | 3000 mg/m² over 3 hours every 12 hours | Days 1–2 |
| L-asparaginase | IV | 10,000 units/m² | Day 4, 11 |
| **Cycle 2** | | | |
| Dexamethasone | PO | 20 mg/m²/day | Days 1–5 |
| 6-mercaptopurine | PO | 100 mg/m² nocte | Days 1–5 |
| Vincristine | IV | 1.5 mg/m² (max 2 mg) | Days 1 and 6 |
| Methotrexate | IV | 1000 mg/m² over 36 hours | Day 1 |
| Cytarabine | IV | 2000 mg/m² over 3 hours every 12 hours | Day 1 |
| Triple intrathecal chemotherapy | IT | — | Day 1 |
| **Cycle 3** | | | |
| Dexamethasone | PO | 20 mg/m²/day | Days 1–5 |
| 6-thioguanine | PO | 100 mg/m² nocte | Days 1–5 |
| Vincristine | IV | 1.5 mg/m² (max 2 mg) | Day 1 |
| Ifosfamide + Mesna | IV | Ifosfamide 4000 mg/m² with Mesna | Days 1–5 |
| Methotrexate | IV | 1000 mg/m² over 36 hours | Day 1 |
| Triple intrathecal chemotherapy | IT | — | Day 1 |
| **In patients with early relapse** | | | |
| **Drug** | **Route** | **Dose** | **Schedule** |
| **Cycle 1** |  |  |  |
| Dexamethasone | PO | 6 mg/m²/day | Days 1–5, 8–13, 15–20 |
| Triple intrathecal chemotherapy | IT | — | Day 1,12 |
| Vincristine | IV | 1.5 mg/m² (max 2 mg) | Day 1, 8 and 13 |
| Cytarabine | IV | 3000 mg/m² over 3 hours every 12 hours | Days 1–2 |
| L-asparaginase | IV | 10,000 units/m² | Day 4, 11 |
| **Cycle 2** |  |  |  |
| Dexamethasone | PO | 20 mg/m²/day | Days 1–5 |
| 6-mercaptopurine | PO | 100 mg/m² nocte | Days 1–5 |
| Vincristine | IV | 1.5 mg/m² (max 2 mg) | Days 1 and 6 |
| Methotrexate | IV | 1000 mg/m² over 36 hours | Day 1 |
| Cytarabine | IV | 2000 mg/m² over 3 hours every 12 hours | Day 1 |
| Triple intrathecal chemotherapy | IT | — | Day 1 |
| **Cycle 3** |  |  |  |
| Dexamethasone | PO | 20 mg/m²/day | Days 1–5, 8-13 |
| 6-thioguanine | PO | 100 mg/m² nocte | Days 1–5, 8-13 |
| Vincristine | IV | 1.5 mg/m² (max 2 mg) | Day 1,8,13 |
| Ifosfamide + Mesna | IV | Ifosfamide 4000 mg/m² with Mesna | Days 1–5 |
| Methotrexate | IV | 1000 mg/m² over 36 hours | Day 1,8 |
| Triple intrathecal chemotherapy | IT | — | Day 1,8 |
| **Cycle 4** |  |  |  |
| Dexamethasone | PO | 20 mg/m²/day | Days 1–5 |
| 6-thioguanine | PO | 100 mg/m² nocte | Days 1–5 |
| Vincristine | IV | 1.5 mg/m² (max 2 mg) | Day 1 |
| Ifosfamide + Mesna | IV | Ifosfamide 4000 mg/m² with Mesna | Days 1–5 |
| Methotrexate | IV | 1000 mg/m² over 36 hours | Day 1 |
| Triple intrathecal chemotherapy | IT | — | Day 1 |
| **Cycle 5** |  |  |  |
| Dexamethasone | PO | 20 mg/m²/day | Days 1–5 |
| 6-mercaptopurine | PO | 100 mg/m² nocte | Days 1–5 |
| Vincristine | IV | 1.5 mg/m² (max 2 mg) | Days 1 and 6 |
| Methotrexate | IV | 1000 mg/m² over 36 hours | Day 1 |
| Cytarabine | IV | 2000 mg/m² over 3 hours every 12 hours | Day 1 |
| Triple intrathecal chemotherapy | IT | — | Day 1 |
| **Cycle 6** |  |  |  |
| Dexamethasone | PO | 20 mg/m²/day | Days 1–5, 8-13 |
| 6-thioguanine | PO | 100 mg/m² nocte | Days 1–5, 8-13 |
| Vincristine | IV | 1.5 mg/m² (max 2 mg) | Day 1,8,13 |
| Ifosfamide + Mesna | IV | Ifosfamide 4000 mg/m² with Mesna | Days 1–5 |
| Methotrexate | IV | 1000 mg/m² over 36 hours | Day 1,8 |
| Triple intrathecal chemotherapy | IT | — | Day 1,8 |
| **Cycle 7 (D24)** |  |  |  |
| 6-thioguanine | PO | 50 mg/m² | Daily for 12 months |
| Methotrexate | IV | 50 mg/m² | Every 2 weeks |
| Etoposide | PO | 50 mg/m²/day | 10 days, starting 6 weeks after maintenance initiation, every 8 weeks |
| **In patients with late relapse** | | | |
| **Drug** | **Route** | **Dose** | **Schedule** |
| **Cycle 1** |  |  |  |
| Dexamethasone | PO | 6 mg/m²/day | Days 1–5, 8–13, 15–20 |
| Triple intrathecal chemotherapy | IT | — | Day 1,12 |
| Vincristine | IV | 1.5 mg/m² (max 2 mg) | Day 1, 8 and 13 |
| Cytarabine | IV | 3000 mg/m² over 3 hours every 12 hours | Days 1–2 |
| L-asparaginase | IV | 10,000 units/m² | Day 4, 11 |
| **Cycle 2** |  |  |  |
| Dexamethasone | PO | 20 mg/m²/day | Days 1–5 |
| 6-mercaptopurine | PO | 100 mg/m² nocte | Days 1–5 |
| Vincristine | IV | 1.5 mg/m² (max 2 mg) | Days 1 and 6 |
| Methotrexate | IV | 1000 mg/m² over 36 hours | Day 1 |
| Cytarabine | IV | 2000 mg/m² over 3 hours every 12 hours | Day 1 |
| Triple intrathecal chemotherapy | IT | — | Day 1 |
| **Cycle 3** |  |  |  |
| Dexamethasone | PO | 20 mg/m²/day | Days 1–5, 8-13 |
| 6-thioguanine | PO | 100 mg/m² nocte | Days 1–5, 8-13 |
| Vincristine | IV | 1.5 mg/m² (max 2 mg) | Day 1,8,13 |
| Ifosfamide + Mesna | IV | Ifosfamide 4000 mg/m² with Mesna | Days 1–5 |
| Methotrexate | IV | 1000 mg/m² over 36 hours | Day 1,8 |
| Triple intrathecal chemotherapy | IT | — | Day 1,8 |
| **Cycle 4** |  |  |  |
| Dexamethasone | PO | 20 mg/m²/day | Days 1–5 |
| 6-thioguanine | PO | 100 mg/m² nocte | Days 1–5 |
| Vincristine | IV | 1.5 mg/m² (max 2 mg) | Day 1 |
| Ifosfamide + Mesna | IV | Ifosfamide 4000 mg/m² with Mesna | Days 1–5 |
| Methotrexate | IV | 1000 mg/m² over 36 hours | Day 1 |
| Triple intrathecal chemotherapy | IT | — | Day 1 |
| **Cycle 5** |  |  |  |
| Dexamethasone | PO | 20 mg/m²/day | Days 1–5 |
| 6-mercaptopurine | PO | 100 mg/m² nocte | Days 1–5 |
| Vincristine | IV | 1.5 mg/m² (max 2 mg) | Days 1 and 6 |
| Methotrexate | IV | 1000 mg/m² over 36 hours | Day 1 |
| Cytarabine | IV | 2000 mg/m² over 3 hours every 12 hours | Day 1 |
| Triple intrathecal chemotherapy | IT | — | Day 1 |
| **Cycle 5 (D12)** |  |  |  |
| 6-thioguanine | PO | 50 mg/m² | Daily for 12 months |
| Methotrexate | IV | 50 mg/m² | Every 2 weeks |

**D24: 24 months of treatment with 6-thioguanine and methotrexate, plus 4 additional oral etoposide pulses at 50 mg/m²/day for 10 days, given once every 8 weeks; D12: 12 months of treatment with 6-thioguanine 50 mg/m² PO daily and methotrexate 50 mg/m² IV every 2 weeks**

**Clinical inputs**

Literature search on MEDLINE (up to 2025) was performed using keywords including “acute”, “lymphoblastic”, “leukemia”, “children”, “standard-risk”, “B-Cell”, “blinatumomab”. The selection criteria of clinical trials were: (1) Reports in English language; (2) pediatric patients with acute lymphoblastic leukemia; and (3) disease-free and overall survival was reported. Preferred studies were meta-analyses and randomized controlled trials. When multiple randomized trials were the source for a single model input, the base-case value was set as a weighted average. The AALL1731 study was a phase 3 randomized trial that evaluated chemotherapy alone and chemotherapy plus blinatumomab in newly diagnosed children with standard-risk B-cell ALL.^1^ The Hong Kong ALL-Relapse 2000 study was a prospective cohort trial (n=56) to evaluate the outcomes (median follow-up 2.7 years) of intensive chemotherapy with risk-based stratification in children with first relapse of acute lymphoblastic leukemia.^2^ The ELIANA study was a global, multicenter, open-label phase II trial that evaluated outcomes of CAR-T in 79 patients with refractory B-cell ALL for a median follow-up of 38.8 months.^5^ The AALL1331 trial was a phase 3 randomized study (n=208) that compared blinatumomab with chemotherapy as post-reinduction therapy in patients with first relapse of B-cell ALL for a median follow-up of 2.9 years.^4^

**Results**

**Sensitivity analysis**

The scatter plots of the increment costs and QALY gained by blinatumomab first-line group and second-line group versus chemotherapy group for patients with high risk and average risk of relapse of relapse were shown in **Supplementary Figure 1A-D**.

For patients with high risk of relapse, the blinatumomab first-line group gained more QALY at higher cost versus chemotherapy group by 0.2560 QALY (95%CI 0.2525-0.2596; p<0.01) and USD 9,522 (95%CI 9,306-9,738 USD; p<0.01) (**Figure S1A**). Blinatumomab second-line group gained higher QALY at higher cost versus chemotherapy group by 0.0901 QALY (95%CI 0.0897-0.0904; p<0.01) and 1,112 USD (95%CI 1,077-1,146 USD; p<0.01) for patients with high risk of relapse (**Figure S1B**).

For patients with average risk of relapse, blinatumomab first-line group gained more QALY at higher cost versus chemotherapy group by 0.2952 QALY (95%CI 0.2932-0.2972; p<0.01) and 14,327 USD (95%CI 14,139-14,516 USD; p<0.01) (**Figure S1C**). Blinatumomab second-line group gained higher QALY at higher cost versus chemotherapy group by 0.0524	QALY (95%CI 0.0521-0.0526; p<0.01) and 763 USD (95%CI 741-785 USD; p<0.01) for patients with average risk of relapse (**Figure S1D**).

**Figure S1. Scatter plots of incremental cost and QALY gained in 10,000 Monte Carlo simulations: (A) Blinatumomab first-line group versus chemotherapy group for patients with high risk of relapse; (B) Blinatumomab second-line group versus chemotherapy group for patients with high risk of relapse; (C) Blinatumomab first-line group versus chemotherapy group for patients with average risk of relapse; (D) Blinatumomab second-line group versus chemotherapy group for patients with average risk of relapse; WTP: willingness-to-pay; QALY: quality-adjusted life-year**

**Reference**

1.	Gupta S, Rau RE, Kairalla JA, et al. Blinatumomab in Standard-Risk B-Cell Acute Lymphoblastic Leukemia in Children. *N Engl J Med*. 2025;392(9):875-891. doi:10.1056/NEJMoa2411680

2.	Leung AW, Lee V, Chiang AK, et al. Prognosis and outcome of relapsed acute lymphoblastic leukemia: a Hong Kong Pediatric Hematology and Oncology Study Group report. *Pediatr Blood Cancer*. 2012;59(3):454-60. doi:10.1002/pbc.24162

3.	Eckert C, von Stackelberg A, Seeger K, et al. Minimal residual disease after induction is the strongest predictor of prognosis in intermediate risk relapsed acute lymphoblastic leukaemia - long-term results of trial ALL-REZ BFM P95/96. *Eur J Cancer*. 2013;49(6):1346-55. doi:10.1016/j.ejca.2012.11.010

4.	Brown PA, Ji L, Xu X, et al. Effect of Postreinduction Therapy Consolidation With Blinatumomab vs Chemotherapy on Disease-Free Survival in Children, Adolescents, and Young Adults With First Relapse of B-Cell Acute Lymphoblastic Leukemia: A Randomized Clinical Trial. *Jama*. 2021;325(9):833-842. doi:10.1001/jama.2021.0669

5.	Laetsch TW, Maude SL, Rives S, et al. Three-Year Update of Tisagenlecleucel in Pediatric and Young Adult Patients With Relapsed/Refractory Acute Lymphoblastic Leukemia in the ELIANA Trial. *J Clin Oncol*. 2023;41(9):1664-1669. doi:10.1200/jco.22.00642
